# Regulation of tillage on grain matter accumulation in maize

**DOI:** 10.3389/fpls.2024.1373624

**Published:** 2024-06-21

**Authors:** Li-Qing Wang, Xiao-Fang Yu, Ju-Lin Gao, Da-Ling Ma, Hong-Yue Liu, Shu-Ping Hu

**Affiliations:** College of Agronomy, Inner Mongolia Agricultural University, Hohhot, China

**Keywords:** spring maize, tillage methods, grain weight, grain filling, grain nutritional quality

## Abstract

**Introduction:**

To address issues related to shallow soil tillage, low soil nutrient content, and single tillage method in maize production in the Western Inner Mongolia Region, this study implemented various tillage and straw return techniques, including strip cultivation, subsoiling, deep tillage, no-tillage, straw incorporation with strip cultivation, straw incorporation with subsoiling, straw incorporation with deep tillage, and straw incorporation with no tillage, while using conventional shallow spinning by farmers as the control.

**Methods:**

We employed Xianyu 696 (XY696) and Ximeng 6 (XM6) as experimental materials to assess maize 100-grains weight, grain filling rate parameters, and grain nutrient quality. This investigation aimed to elucidate how tillage and straw return influence the accumulation of grain material in different maize varieties.

**Results and discussion:**

The results indicated that proper implementation of tillage and straw return had a significant impact on the 100-grains weight of both varieties. In comparison to CK (farmer’s rotary rotation), the most notable rise in 100-grains weight was observed under the DPR treatment (straw incorporation with deep tillage), with a maximum increase of 4.84% for XY696 and 6.28% for XM6. The proper implementation of tillage and straw return in the field resulted in discernible differences in the stages of improving the grain filling rates of different maize varieties. Specifically, XY696 showed a predominant increase in the filling rate during the early stage (V1), while XM6 exhibited an increase in the filling rates during the middle and late stages (V2 and V3). In comparison to CK, V1 increased by 1.54% to 27.56% in XY696, and V2 and V3 increased by 0.41% to 10.42% in XM6 under various tillage and straw return practices. The proper implementation of tillage and straw return had a significant impact on the nutritional quality of the grains in each variety. In comparison to CK, the DPR treatment resulted in the most pronounced decrease in the soluble sugar content of grains by 25.43% and the greatest increase in the crude fat content of grains by 9.67%.

**Conclusion:**

Ultimately, the proper implementation of soil tillage and straw return facilitated an increase in grain crude fat content and significantly boosted grain weight by improving the grouting rate parameters at all stages for various maize varieties. Additionally, the utilization of DPR treatment proved to be more effective. Overall, DPR is the most promising strategy to improve maize yield and the nutritional quality of grain in the long term in the Western Inner Mongolia Region.

## 1 Introduction

The Inner Mongolia Autonomous Region serves as a crucial national grain production base, encompassing 11.5 million hectares of arable land, which represents approximately 9.0% of the country’s total arable land. According to statistical data, the aggregate production of grain crops in Inner Mongolia amounted to 3.66 million tonnes in 2021, positioning it as the sixth largest producer in the country (Chen et al., 2021). Nevertheless, the occurrence of issues including diminished soil fertility from prolonged continuous cropping, autotoxicity resulting from plant root secretions, and heightened susceptibility to microbial diseases diminishes crop nutrient absorption and leads to decreased crop yield per unit area (Rubio et al., 2021; Xing et al., 2022), thereby significantly constraining the region’s agricultural sustainability. Hence, it is imperative to investigate rational and practical plowing techniques in the region to enhance the soil quality and fertility, thereby safeguarding the national food security.

Prolonged reliance on traditional shallow rotary plowing in farmland in the Western Inner Mongolia Region has led to the formation of a compacted soil tillage layer, elevated soil bulk density, constrained root growth, and hindered nutrient and water absorption, thereby constraining crop growth and yield potential (Liu et al., 2022a; Yu et al., 2023). Deep plowing, no tillage, strip tillage, and other plowing techniques constitute crucial practices in agricultural production. These methods have a direct influence on achieving high and consistent crop yields and promoting sustainable development through alterations in soil structure and physico-chemical properties (Abu-Hamdeh, 2003; Zhang et al., 2021; Wang et al., 2022). Subsoiling enhances soil porosity, facilitating deep rooting and nutrient uptake without causing soil compaction or excessive drying. Deep tilling breaks up the soil crusts and improves the soil permeability, thereby enhancing the exchange of nutrients and gases between soil layers. It also promotes the decomposition of deeply buried straw, resulting in increased soil fertility and the reduction of pests and diseases (He et al., 2021). Conservation tillage methods such as no tillage and minimum tillage mitigate wind and water erosion, reduce evaporation, and contribute to enhancing soil fertility, cost savings, and overall efficiency in land cultivation (Yuan et al., 2018; Pecci et al., 2021). Nevertheless, no tillage contributes to increased soil bulk density and compactness (Blanco-Canqui et al., 2022), lowered soil temperature, and constrained the growth of the maize root system, consequently impacting the nutrient uptake in the deeper soil layers (Li et al., 2022a). Strip rotary tillage improves the photosynthetic efficiency of plant leaves during the late filling stage, enabling enhanced dry matter translocation to the grains and stimulating post-anthesis dry matter accumulation (Chu et al., 2016). Therefore, improvement in soil structure, enhanced crop nutrient uptake, increased grain dry matter accumulation, and enhanced crop yields can be achieved only through the adoption of reasonable plowing measures (Zhai et al., 2021).

The practice of returning straw to the field is an effective method for comprehensively utilizing straw and plays a crucial role in regulating the soil structure, enhancing the crop root structure and function, and safeguarding the ecological environment (Zhang et al., 2014; Qin et al., 2015). Returning straw to the field reduces soil bulk density and enhances total soil porosity, significantly improving soil aeration and facilitating the deeper penetration of crop roots (Yao et al., 2015; Zhang et al., 2015). Upon returning the straw to the field, the decomposition and release of organic matter from the straw enhance the soil’s organic matter content and lead to increased levels of available nitrogen, phosphorus, and potassium (Tan et al., 2015). Variations in climatic conditions, soil properties, and tillage management necessitate a judicious combination of straw return methods and tillage practices to notably enhance the efficiency of soil nutrient utilization by the crop root system. Most of the previous studies have focused on the effects of tillage practices on soil and maize plants—for instance, no tillage and straw return safeguard soil structure and protect organic carbon aggregates from microbial degradation, leading to augmented storage of soil organic carbon (SOC), minimized SOC mineralization, and enhanced crop biomass and yield (Sainju et al., 2009; Xu et al., 2019a). Additionally, it has been suggested that deep tilling of straw can effectively address the issues related to delayed straw decomposition and impediments to the emergence of maize seedlings during the process of field straw return. At the same time, it can significantly improve the characteristics of soil structure and increase the soil nutrients (Xu et al., 2019b; Jin et al., 2020; Zhang et al., 2023). Studies investigating the impact of straw return on yield enhancement have found that deep plowing of returned straw indirectly affects maize yield and its components by directly and preferentially influencing the levels of total and readily available nitrogen in the soil (Wang et al., 2023a). In the realm of intensive maize cultivation, grain weight plays a critical role in enhancing maize yields, with a direct correlation to grain filling characteristics that are significantly impacted by tillage techniques and straw management (Shao et al., 2016; Ren et al., 2021).

A favorable soil micro-environment enhances organic carbon mineralization and stable soil nutrient supply and thus also contributes to improve the grain yield. Therefore, improving soil structure and facilitating maize nutrient uptake through the incorporation of straw into the field, along with appropriate plowing practices, hold a crucial significance for the sustainable utilization of farmland in the Western Inner Mongolia Region. Prior research has extensively studied the impacts of various tillage methodologies on soil conditions and their subsequent ecological advantages. However, comparatively limited studies have endeavored to elucidate the underlying mechanisms of yield variations seen across these tillage techniques from the standpoint of grain filling. Accordingly, this experiment employed a combination of continuous positional tillage and straw return methods in the early stage of the trial to examine the evolving patterns of maize grain filling traits, aiming to establish a theoretical framework for optimizing soil tillage, rational straw resource utilization, and maximizing maize yield in the Western Inner Mongolia Region. The present study hypothesized that DPR (straw incorporation with deep tillage) could effectively ensure the increase of grain weight and the improvement of grain nutritional quality during maize cultivation in Western Inner Mongolia. The objectives of this study were (i) to determine the most suitable tillage practices based on changes in 100-grains weight and grain nutrient quality, (ii) to understand the characteristics of grain filling and the pattern of change in filling rate parameters under different tillage practices, and (iii) to study the relationship between different components of grain nutrient quality and filling rate parameters, elucidating the potential mechanism by which these components respond to 100-grains weight.

## 2 Materials and methods

### 2.1 Description of research location

**Figure 1:**
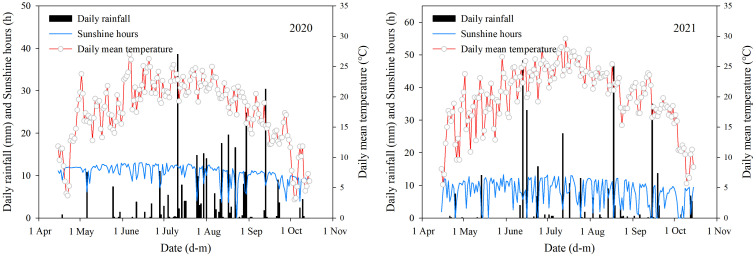
Main meteorological factors during the growing period in the experimental area.

The field experiments took place at the Tumoteyou Qi Experimental Station of Inner Mongolia Agricultural University (40°33′ N, 110°31′ E) in 2020 and 2021. The tillage method testing platform was constructed in autumn 2017 and repeated each year for the previous year’s tillage practices. The preceding crop was maize, and the soil type was sandy loam. The nutrient data for the plowed ground (0–30 cm soil layer) before sowing can be found in **Table 1**. **Figure 1** illustrates the primary meteorological factors that influenced maize growth during the study period. During the maize growing period in 2020 and 2021, the average daily temperature was 18.92°C and 21.19°C, the total rainfall was 328.40 and 310.30 mm, and the total sunshine hours was 1,761.58 and 1,362.02 h, respectively.

**Table 1:** Soil nutrients under different tillage methods in 2020 and 2021.

Tillage method	Year	Alkali-hydrolysable N	Available P	Available K	Organic matter
		(mg kg^-1^)	(mg kg^-1^)	(mg kg^-1^)	(g kg^-1^)
Farmer rotary tillage (CK)	2020	53.44	2.52	64.79	17.36
	2021	53.07	2.57	66.07	17.44
Strip cultivation (SC)	2020	53.20	3.35	71.54	20.13
	2021	54.59	3.34	72.38	19.67
Subsoiling (SS(	2020	55.57	3.01	83.88	22.35
	2021	57.91	3.10	85.81	22.50
Deep tillage (DP)	2020	61.27	2.68	89.98	22.52
	2021	62.58	2.78	90.11	22.80
No-tillage (NT)	2020	65.09	4.42	79.86	20.96
	2021	66.16	4.47	84.26	20.67
Straw incorporation with strip cultivation (SCR)	2020	62.44	3.84	85.85	22.71
	2021	63.12	3.91	87.90	23.18
Straw incorporation with subsoiling (SSR)	2020	72.84	3.32	94.45	23.76
	2021	74.16	3.49	99.02	23.94
Straw incorporation with deep tillage (DPR)	2020	66.77	3.65	113.18	24.86
	2021	70.34	3.83	117.03	25.37
Straw incorporation with no-tillage (NTR)	2020	74.11	4.60	84.28	24.06
	2021	72.90	4.67	89.42	23.98

### 2.2 Experimental design

A split-zone experimental design was implemented, where the plowing method was applied in the central zone. The farmer’s rotary rotation served as the control (CK). Eight treatments were established for comparison, comprising subsoiling (SS), deep tillage (DP), strip cultivation (SC), no tillage (NT), straw incorporation with strip cultivation (SCR), straw incorporation with subsoiling (SSR), straw incorporation with deep tillage (DPR), and straw incorporation with no tillage (NTR). There was a total of nine tillage treatments. In combination with the tillage methods, two different densely tolerant maize varieties (Xianyu 696 and Ximeng 6) were used, providing a total of 18 treatments, each with three replicates. Xianyu 696 (XY696) and Ximeng 6 (XM6) were provided by Dunhuang Seed Pioneer Variety Co. Ltd., and Inner Mongolia Simon Seed Co. Ltd., which have a fertility period of 125 and 130 days, respectively. Consequently, the experiment consisted of a total of 54 plots, each with an area of 65 m × 6 m. The tillage methods and varieties were assigned to the main plot and subplots, respectively. In subplots, varieties were randomly planted with a row spacing of 60 cm. The nine methods of cultivation are shown in **Table 2**.

**Table 2:** Tillage methods’ operating procedures.

Abridge	Tillage methods	Depth (cm)	Methods of tillage practices
CK	Farmer’s rotary rotation	15	The straw was removed from the field after being mechanically harvested in autumn, shallowly tilled using a rotary tiller (1BX-4.0, Xinjiang Xinyan Mushin Technology Co., China) in the following spring, and then mechanically sown using a planter (Optina SX-12, Kverneland Group, German).
SC	Strip cultivation	30	The straw had been removed from the field after mechanical harvesting in autumn and subsequently mechanically sown in the following spring using a strip-deep rotary planter (none, Beijing Hehuinong Agricultural Resources Co., China).
SS	Subsoiling	35	After the mechanical harvest in autumn, the straw was removed from the field. Subsequently, subsoiling was conducted using a subsoiler (Sub-tiller, HE-VA Group, Denmark), followed by shallow cultivation with a rotary tiller (1BX-4.0, Xinjiang Xinyan Mushin Technology Co., China). Finally, mechanical (Optina SX-12, Kverneland Group, German) seeding took place in the following spring.
DP	Deep tillage	45	After the mechanical harvest in autumn, the straw was removed from the field. Subsequently, deep tillage was conducted using a deep turner (ILFT550, Xinjiang Xinyan Mushin Technology Co., China), followed by shallow cultivation with a rotary tiller (1BX-4.0, Xinjiang Xinyan Mushin Technology Co., China). Finally, mechanical (Optina SX-12, Kverneland Group, German) seeding took place in the following spring.
NT	No tillage		The straw was removed from the field after mechanical harvesting in autumn and then mechanically (1006NT, Grean Plant Group, USA) sown in the following spring using a no-till planter.
SCR	Straw incorporation with strip cultivation	30	The straw was mechanically (4YZB-5AS, Xinjiang Xinyan Mushin Technology Co., China) harvested in autumn, fully crushed, and returned to the field by covering it on the soil surface. It was then sown in the following spring using a strip deep rotary planter (none, Beijing Hehuinong Agricultural Resources Co., China).
SSR	Straw incorporation with subsoiling	35	After having been mechanically (4YZB-5AS, Xinjiang Xinyan Mushin Technology Co., China) harvested in autumn, the straw was thoroughly crushed and applied as a soil cover to be returned to the field. Simultaneously, subsoiling was performed using a subsoiler (Sub-tiller, HE-VA Group, Denmark), followed by incorporating the straw into the soil with a rotary tiller (1BX-4.0, Xinjiang Xinyan Mushin Technology Co., China). Finally, during the subsequent spring season, seeds were sown utilizing a precision seeder (Optina SX-12, Kverneland Group, German).
DPR	Straw incorporation with deep tillage	45	After the mechanical (4YZB-5AS, Xinjiang Xinyan Mushin Technology Co., China) harvest in autumn, the straw was thoroughly crushed and applied as a soil cover to be returned to the field. Simultaneously, a deep tiller machine (ILFT550, Xinjiang Xinyan Mushin Technology Co., China) was employed to incorporate the straw into the soil, followed by rotary tilling using a rotary tiller (1BX-4.0, Xinjiang Xinyan Mushin Technology Co., China). Finally, in the subsequent spring season, seeds were sown utilizing a planter (Optina SX-12, Kverneland Group, German).
NTR	Straw incorporation with no-tillage		The straw was mechanically (4YZB-5AS, Xinjiang Xinyan Mushin Technology Co., China) harvested in autumn, thoroughly crushed, and covered on the soil surface to be returned to the field. It was then seeded in the following spring using a no-till planter (1006NT, Grean Plant Group, USA).

### 2.3 Crop husbandry

In 2020, maize was sown on April 25 and harvested on October 6; in 2021, maize was sown on 22 April and harvested on 7 October. The planting density was 82,500 plants ha^-1^. Basal fertilization included the application of ammonium phosphate dibasic and potassium sulfate before seeding. Ammonium dihydrogen phosphate (N 18%; P_2_O_5_ 46%) was applied at a rate of 375 kg ha^-1^, and potassium sulfate (K_2_O 51%) was applied at a rate of 150 kg ha^-1^. Urea (N, 46%) is utilized as a supplementary fertilizer with application rates of 30% at V6 (sixth leaf), 60% at V12 (12th leaf), and 10% at R2 (blister), with an overall rate of 345 kg ha^-1^. Annually, during the entire growing cycle of maize, the following fertilizer quantities were utilized: 226.2 kg ha^-1^ of pure nitrogen (N), 76.5 kg ha^-1^ of potassium oxide (K_2_O), and 172.5 kg ha^-1^ of phosphorus pentoxide (P_2_O_5_). Drip irrigation was administered four times throughout the growth stages: at V6, V12, R1 (silking), and R2. Each irrigation event encompassed an area of 750 m^3^ ha^-1^. All other management practices conformed to the standard procedures used in large-scale farming operations.

### 2.4 Measurement

#### 2.4.1 Determination of 100-grains weight

At physiological maturity, 10 ears were randomly chosen from each plot and air-dried, and then 100 kernels were collected from the middle of each ear, weighed, and subsequently standardized to obtain the 100-grains weight at 14% moisture content.

#### 2.4.2 Determination of grain filling rate

Starting from 15 days post-pollination, samples were collected at 5-day intervals until the end of the filling period. At each sampling point, three ears per plot were collected, and 100 grains were sampled from the middle of each ear. The grains were then weighed, placed into an oven at 105°C for 30 min, dried at 60°C until reaching a constant weight, and subsequently re-weighed.


Filling rate(Gmean)=(W1−W2)D

where W1 = 100-grains dry weight of the current sample (g), W2 = 100-grains dry weight of the previous sample (g), and *D* = number of days between samples (d).

#### 2.4.3 Determination of grain filling characteristics

Since the process of grain dry matter accumulation adheres to the “S” type growth curve, we used the logistic equation to model the process of grain dry matter accumulation. A logistic equation was used to fit the grain filling process, calculate grain filling characteristic parameters, and analyze grain filling growth. The logistic equation was as follows:


$$W=A/\left(1+Be^{-Ct}\right)$$

In the equation above, *t* is the number of days after flowering (blooming day t0 = 0), *W* is the 100-grains weight after flowering (grain weight on flowering day = w0), *A* is the theoretical maximum 100-grains weight, and B and C are shape parameters. The filling parameters were derived from the first and second derivatives of the equation.

t1 (the start date of the filling peak period) = (lnB − 1.317)/C, corresponding to the grain weight (w1) at this time: w1=*A*/(1 + *Be* ^−*Ct*1^)

t2 (the end date of the filling peak period) = (lnB + 1.317)/C, corresponding to the grain weight (w2) at this time: w2=*A*/(1 + *Be* ^−*Ct*2^)

t3 (the grain weight reaches 99% after flowering, the effective filling period) = (lnB + 4.59512)/C, corresponding to the grain weight (w3) at this time.

The filling duration of the gradually increasing period was calculated as T1 = t1− t0. The increase in grain weight during the rapidly increasing period was calculated as w1 = W1− W0. The mean filling rate of the gradually increasing period was calculated as V1 = w1/T1.

The filling duration of the rapidly increasing period was calculated as T2 = t2− t1. The increase in grain weight during the rapidly increasing period was calculated as w2 = W2 − W1. The mean filling rate of the rapidly increasing stage was calculated as V2 = w2/T2.

The filling duration of the slowly increasing period was calculated as T3 = t3− t2. The increase in grain weight of the slowly increasing period was calculated as w3 = W3− W2. The mean filling rate of the slowly increasing stage was calculated as V3 = w3/T3.

#### 2.4.4 Determination of the nutrient quality components of grains

At physiological maturity, a representative cob was chosen, and the central grains of the cob were oven-dried at 105°C for 30 min, followed by drying to a constant weight at 60°C, and then crushed for measurement. The total nitrogen content of the grains was determined by employing the semi-micro Kjeldahl method, and the crude protein content was calculated by multiplying the total nitrogen content by a factor of 6.25. The determination of crude fat content utilized the Soxhlet extraction–residue method (Atif and Perveen, 2023), and the total starch and total soluble sugar content were assessed following the method described by Yu et al. (2022).

### 2.5 Statistical analysis

The data were collected and organized using Microsoft Excel 2019 (Microsoft, Inc., Redmond, WA, USA). Data analysis was conducted using SAS 9.4 (SAS Institute Inc., Raleigh, NC, USA) for variance analysis, correlation analysis, stepwise regression, and principal component analysis. A two-way ANOVA was carried out to explore the impact of tillage methods on the 100-grains weight and nutritional quality components across two varieties. The significance test was performed using LSD (least significant difference) at a significance level of 5%. Correlation analysis was conducted using the Pearson correlation method. Sigmaplot 12.5 (Systat Software, Inc., San Jose, CA, USA) and Origin 2021 (OriginLab Corp., Northampton, MA, USA) were utilized to generate graphs.

## 3 Results

### 3.1 Regulatory effects of tillage methods on 100-grains weight of two maize varieties

The ANOVA results (**Table 3**) indicated highly significant differences in the 100-grains weight among tillage methods or varieties in 2020 (*p*< 0.01). Similarly, in 2021, there were highly significant differences in 100-grains weight among tillage methods or varieties, and significant differences among tillage methods × varieties were observed (*p*< 0.05).

**Table 3:** Variance analysis of the effect of tillage method and variety on the 100-grains weight.

Sources of variation	2020 year	2021 year
Tillage method (M)	13.08**	19.21**
Error I MS	0.010	0.170
Varieties(V)	113.73**	408.64**
M × V	0.02	2.94*
Error II MS	0.009	0.090

*Significant at p< 0.05.

**Significant at p< 0.01.

**Figure 2:**
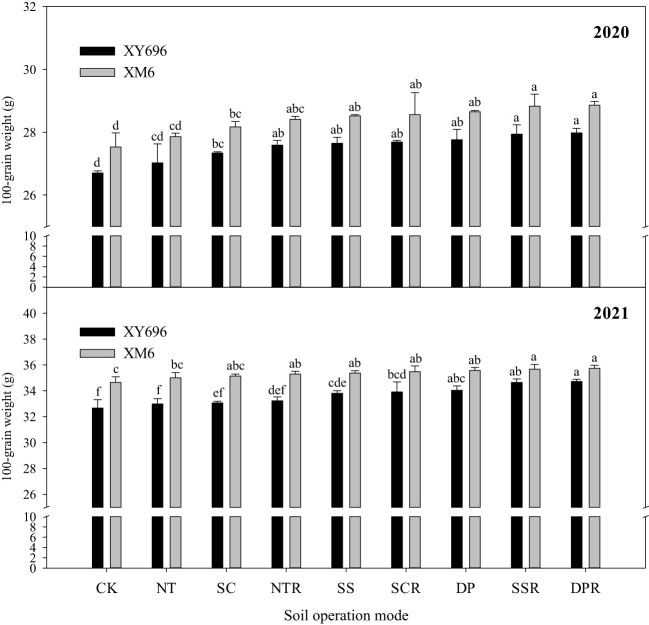
Effects of tillage methods on the 100-grains weight of two maize varieties. Different lowercase letters indicate significant differences at the 0.05 level between different tillage methods for the same variety.

The 100-grains weight of XM6 exceeded that of XY696 under the farmer’s rotary tillage treatment (CK) by 3.11% in 2020 and 6.05% in 2021 (**Figure 2**). Various tillage and straw return methods led to changes in the 100-grains weight of each maize variety, with straw incorporation with subsoiling (SSR) and straw incorporation with deep tillage (DPR) showing superior performance. In 2020, the 100-grains weight of XY696 significantly increased by 4.66% and 4.80% under SSR and DPR treatments, respectively, compared to CK; similarly, XM6’s weight increased by 4.73% and 4.84%. In 2021, under similar contrast conditions, the 100-grains weight of XY696 saw a significant increase by 6.05% and 6.28%, while XM6’s weight increased by 2.95% and 3.15%.

### 3.2 Regulatory effects of tillage methods on the grain filling rate of two maize varieties

**Figure 3:**
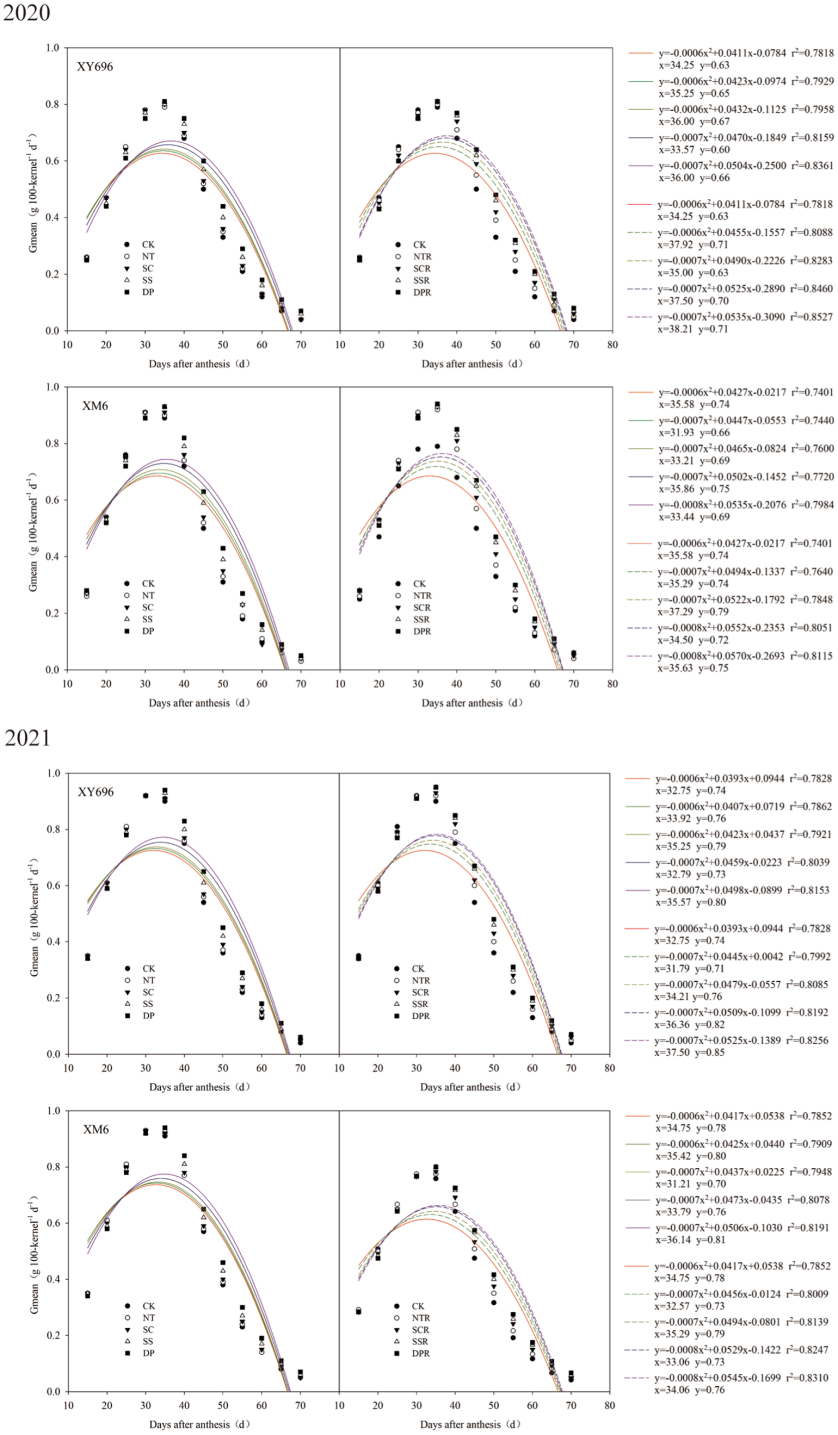
Effects of tillage methods on the grain filling rate of two maize varieties.

**Figure 3** illustrates a quadratic trend in the grain filling rate under each treatment, characterized by an initial increase followed by a decrease. In CK, the peak grouting rate of XM6 surpassed that of XY696, and the time of peak grouting rate occurrence was also delayed. In comparison to CK, the peak grouting rate (*y*) and its occurrence time (*x*) differed across the other treatments. The peak grouting rate of XY696 under SSR and DPR treatments increased by 9.49% and 11.57% in 2020 and 11.01% and 14.50% in 2021, and the time of occurrence of peak grouting rate was prolonged by 11.18% and 14.04% in 2020 and 10.50% and 14.57% in 2021, in turn, as compared to CK treatment. Under the same comparison conditions, the peak grouting rate of XM6 changed sequentially by -3.04% and 0.12% in 2020 and -4.86% and -1.98% in 2021, and the time of peak grouting rate occurrence changed sequentially by -2.86% and 1.09% in 2020 and -5.91% and -2.57% in 2021.

### 3.3 Regulatory effects of tillage methods on the parameters of grain filling rate in two maize varieties

**Figure 4:**
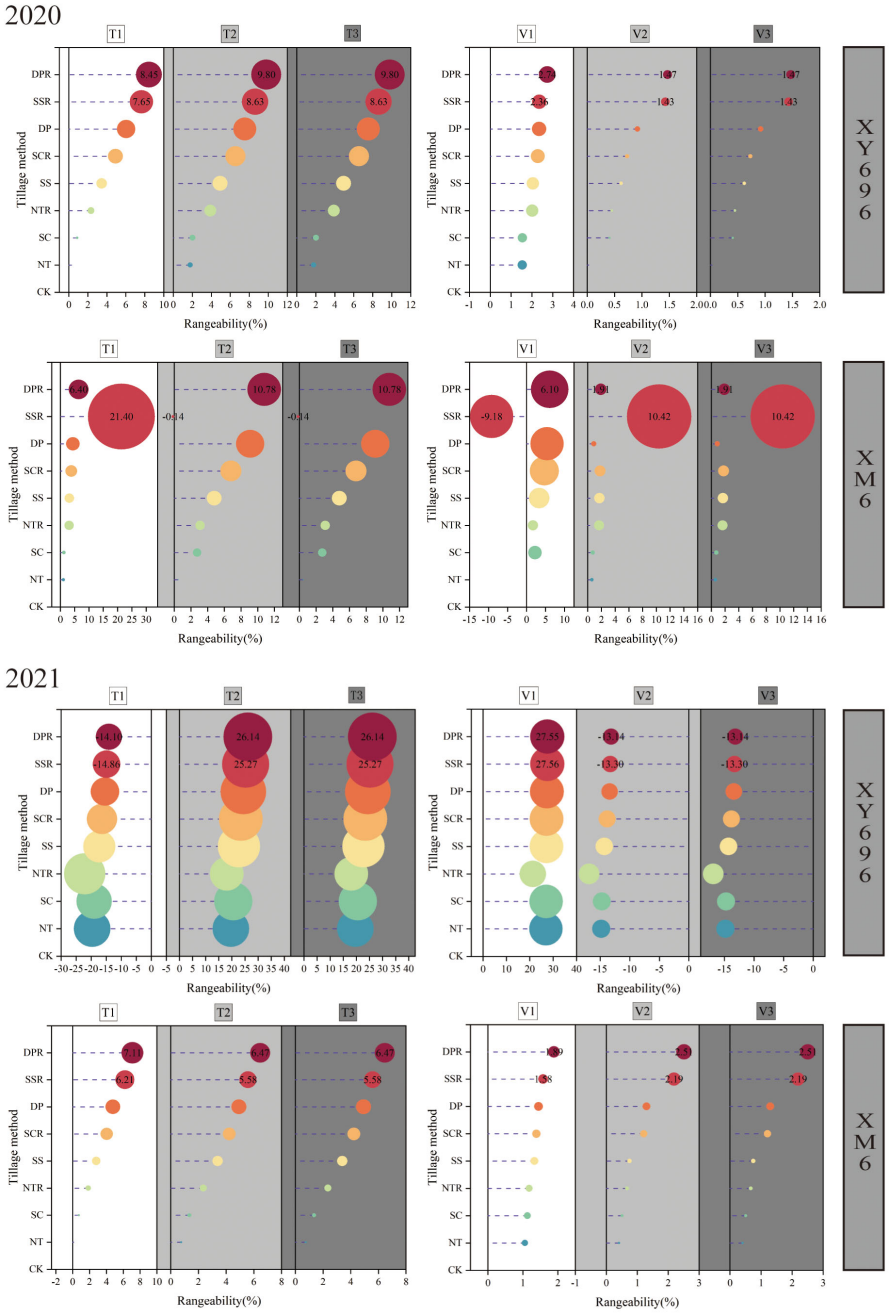
Effects of tillage methods on the grain filling rate parameters of two maize varieties.

The logistic curve equation divided maize grain filling into three periods: gradual increase, fast increase, and slow increase. The duration of filling in each stage followed the order: slow increase period > fast increase period > gradual increase period. The average filling rate in each stage followed the sequence: fast increase period > gradual increase period > slow increase period (**Figure 4**). In the conditions of farmers’ shallow spinning (CK), XM6 exhibited a lower asymptotic grouting duration (T1) and a higher asymptotic grouting rate (V1) than XY696. Nevertheless, the pattern of change in grouting duration (T2, T3) and grouting rate (V2, V3) between varieties during fast and slow growth periods was not consistent across the two growing seasons.

Regarding grouting rate, V1 of XY696 exhibited an increase under SSR and DPR treatments compared to CK: 2.36% and 2.74% in 2020 and 27.56% and 27.55% in 2021, while V2 changed by 1.43% and 1.47% in 2020 and -13.30% and -13.14% in 2021, and V3 changed by 1.43% and 1.47% in 2020 and -13.30% and -13.14% in 2021. In similar contrasting conditions, XM6 exhibited a preference for adjustments in mid- to late-stage grouting rates (V2 and V3), exemplified by V2 increasing by 10.42% and 1.91% in 2020 and by 2.19% and 2.51% in 2021 and V3 exhibiting growth by 10.42% and 1.91% in 2020, followed by 2.19% and 2.51% in 2021. Concerning grouting duration, in comparison to CK, the T1 of XY696 under each SSR and DPR treatment exhibited changes of 7.65% and 8.45% in 2020, and -14.86% and -14.10% in 2021, while T2 increased by 8.63% and 9.80% in 2020 and 25.27% and 26.14% in 2021, and T3 increased by 8.63% and 9.80% in 2020 and 25.27% and 26.14% in 2021 sequentially. Under similar comparison conditions, XM6 demonstrated a preference for alterations in the pre-grouting duration, exemplified by T1 increasing sequentially by 21.40% and 6.40% in 2020 and by 6.21% and 7.11% in 2021.

### 3.4 Regulatory effects of tillage methods on the nutritional quality of grains of two maize varieties

The ANOVA results (**Tables 4**, **5**) indicated a highly significant difference in grain crude fat content among tillage methods and a highly significant difference in grain total soluble sugar content among tillage methods, varieties, or their interactions in 2020. In 2021, highly significant differences were observed in grain crude fat content between the tillage method and varieties and in grain total soluble sugar content among tillage methods and varieties.

**Table 4:** Variance analysis of the effect of tillage method and variety on the nutritional quality of maize grains in 2020.

Sources of variation	Crude protein content	Total starch content	Crude fat content	Total soluble sugar content
Tillage method (M)	0.46	1.54	13.19**	118.32**
Error I MS	0.012	0.613	0.007	0.017
Varieties(V)	4.25	1.12	0.22	551.63**
M × V	0.01	0.02	1.47	8.01**
Error II MS	0.007	0.884	0.005	0.024

**Significant at p< 0.01.

**Table 5:** Variance analysis of the effect of tillage method and variety on the nutritional quality of maize grains in 2021.

Sources of variation	Crude protein content	Total starch content	Crude fat content	Total soluble sugar content
Tillage method (M)	0.34	1.82	27.75**	24.43**
Error I MS	0.008	0.709	0.005	0.085
Varieties (V)	0.25	0.80	63.08**	222.86**
M × V	0.00	0.01	2.12	0.18
Error II MS	0.013	0.855	0.005	0.123

**Significant at p< 0.01.

**Figure 5:**
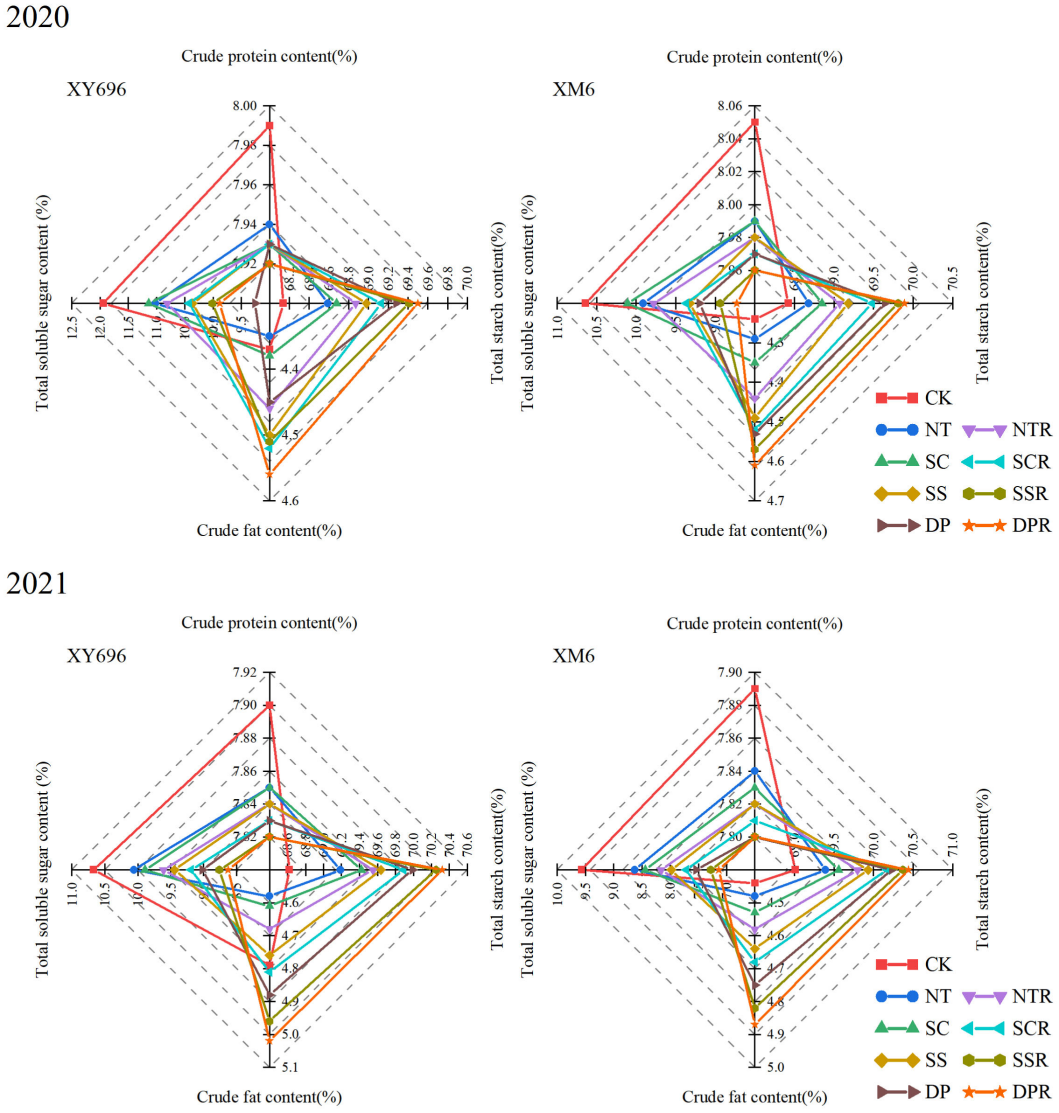
Effects of tillage methods on the grain nutrient quality of two maize varieties.

From **Figure 5**, it can be seen that, compared to CK, the changes in crude protein and total starch content of the grains under the other tillage treatments are relatively small, while there are significant differences in crude fat and soluble sugar content of the grains. In 2020, compared to CK, the crude fat content of XY696 grains only significantly increased under the DPR treatment (4.34%); the soluble total sugar content of the grains showed the largest decrease under the deep tillage (DP) and DPR treatments (22.42% and 17.23%, respectively). Under the same comparative conditions, the crude fat content of XM6 grains showed the largest increase under the SSR and DPR treatments (7.81% and 8.87%, respectively); the soluble total sugar content of the grains also showed the largest decrease under the SSR and DPR treatments (16.04% and 18.01%, respectively). In 2021, compared to CK, the crude fat content of XY696 grains significantly increased under the SSR and DPR treatments (3.49% and 4.71%, respectively); the soluble total sugar content of the grains showed the largest decrease under the SSR and DPR treatments (17.93% and 19.18%, respectively). Under the same comparative conditions, the crude fat content of XM6 grains showed the largest increase under the SSR and DPR treatments (8.52% and 9.67%, respectively); the soluble total sugar content of the grains also showed the largest decrease under the SSR and DPR treatments (23.96% and 25.43%, respectively).

### 3.5 Correlation between 100-grains dry weight and parameters of grain filling rate

**Figure 6:**
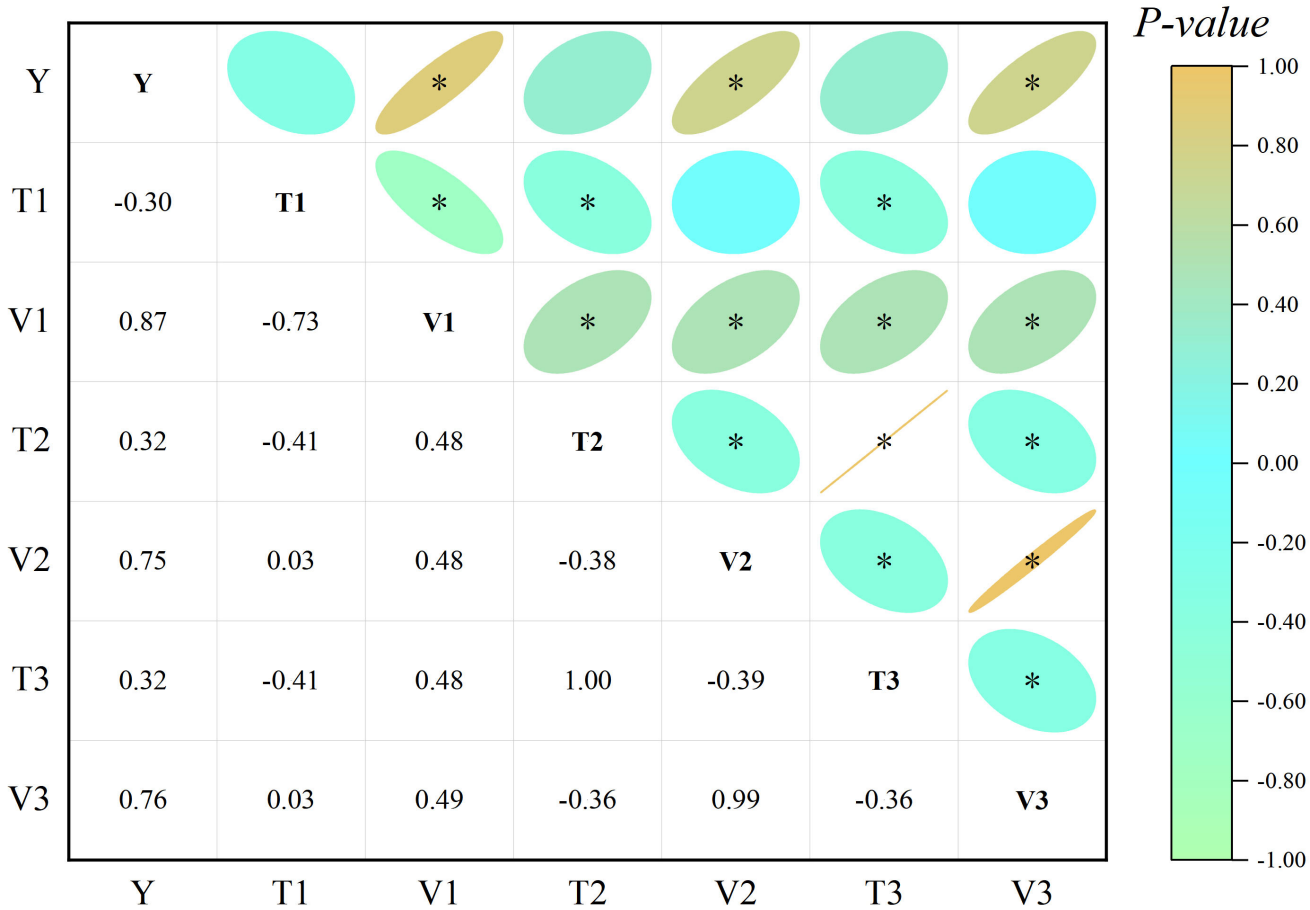
Correlation between the 100-grains dry weight and grain filling rate parameters. Y represents the dry weight of 100 grains. *, significant at *p*< 0.05.

The correlation analyses, as depicted in **Figure 6**, revealed highly significant, positive correlations (0.87, 0.75, and 0.76, *p*< 0.01) between the grain dry weight and the rate of grain filling at each stage (V1–V3), with relatively smaller correlations observed with the duration of grain filling at each stage. Significant positive correlations (0.48, 0.49, 0.48, and 0.49, *p*< 0.01) were observed between V1 and V2, T2, V3, and T3. Additionally, V2 exhibited a significant negative correlation (-0.39, *p*< 0.05) with T3 and a highly significant positive correlation (0.99, *p*< 0.01) with V3.

### 3.6 Correlation between nutritive quality components and 100-grains dry weight of grains

**Figure 7:**
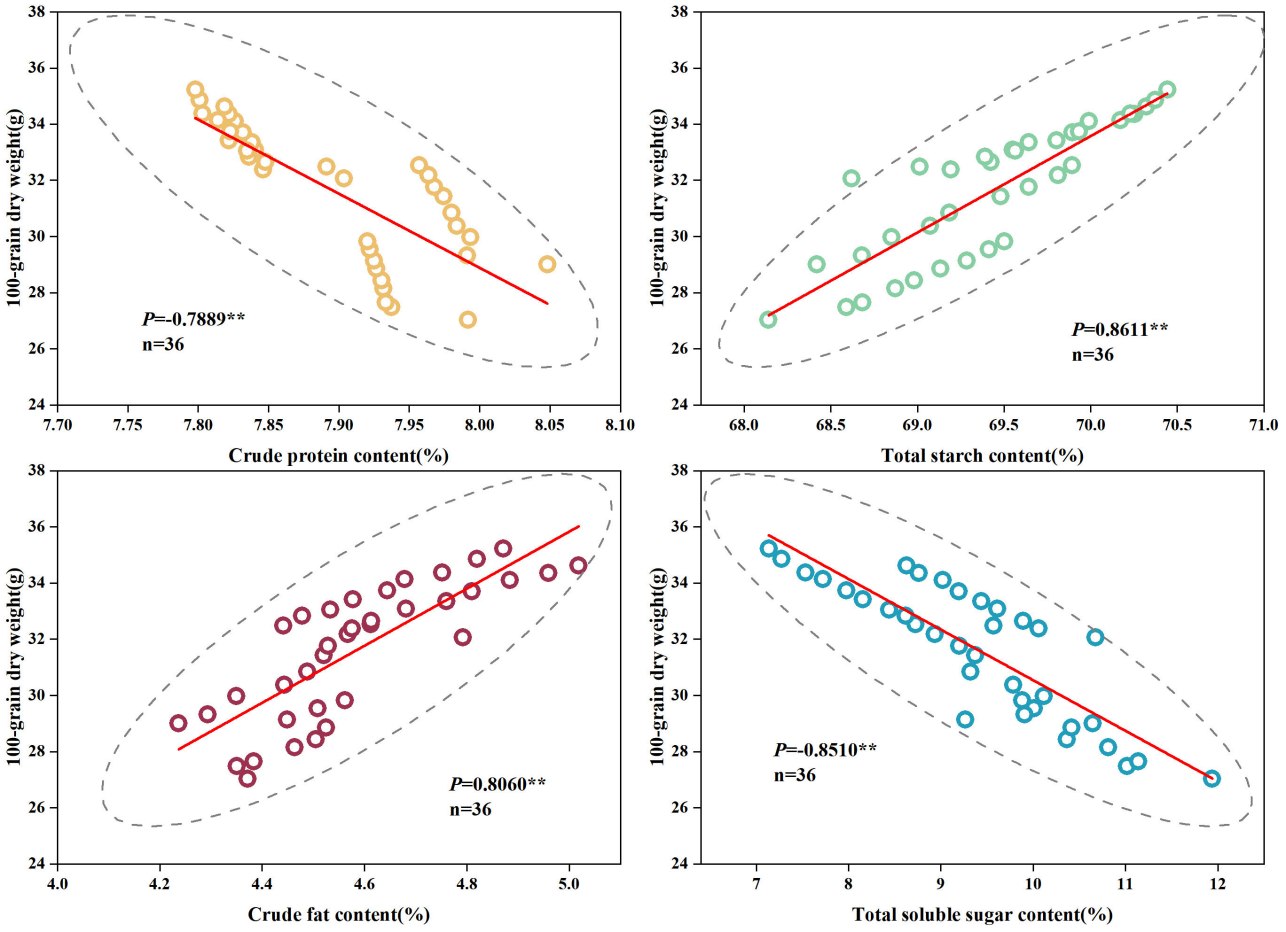
Correlation analysis between the nutrient quality components of grain and dry weight of 100-grains. **, significant at *p*< 0.01.

The results of the correlation analysis depicted in **Figure 7** indicate highly significant negative correlations between the dry weight of grain and the content of grain crude protein and total soluble sugar (-0.79 and -0.85, *p*< 0.01) as well as highly significant positive correlations with total starch and crude fat content of grain (0.86 and 0.81, *p*< 0.01).

### 3.7 Principal component analysis of grain filling rate parameters and grain nutritional quality

**Figure 8:**
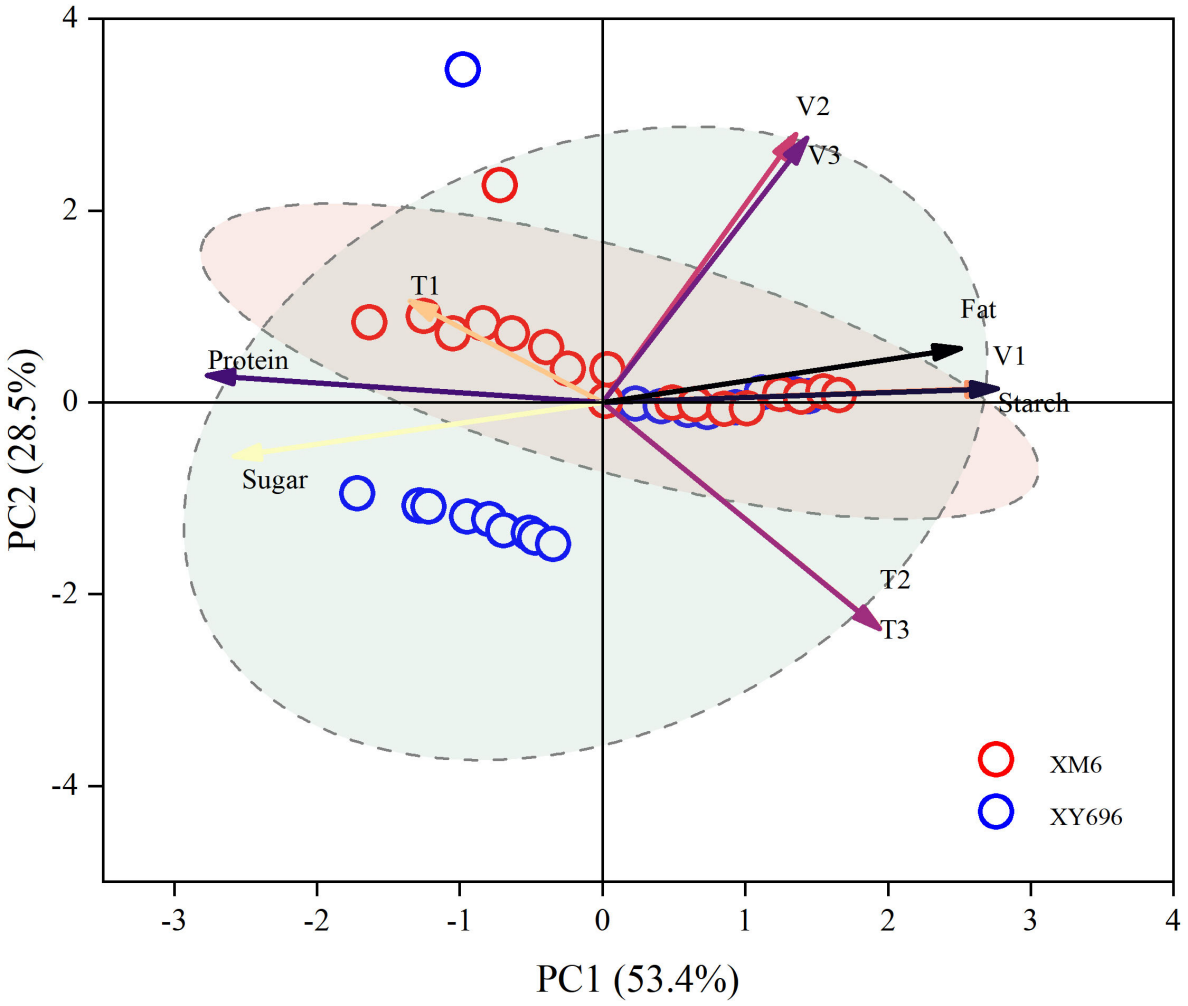
Principal component analysis of grain filling rate and grain nutrient quality. “Protein” stands for crude protein content in grains, “Starch” stands for total starch content in grains, “Fat” stands for crude fat content in grains, and “Sugar” stands for total soluble sugar content in grains.

The principal component analysis accounted for 81.9% of the total variance, with 53.4% contributed by PCA1 and 28.5% by PCA2 (**Figure 8**). Notably, the rate of grouting in the tapering stage (V1) exhibited a highly significant and positive correlation with the total starch content of the grain (Starch), whereas the duration of grouting in the fast-growing stage (T2) showed a highly significant and positive correlation with the duration of grouting in the slow-growing stage (T3), resulting in a substantial overlap in the principal component loadings. The contributions of grain crude fat content (Fat), V1, Starch, grain crude protein content (Protein), grain total soluble sugar content (Sugar), and the duration of asymptotic grouting (T1) were higher in the direction of PCA1. Conversely, toward PCA2, the fast-accelerating rate of grouting (V2), slow-accelerating rate of grouting (V3), T2, and T3 made more significant contributions. In relation to grouting rate, the correlations of the grouting rate parameters (V1, V2, and V3) at each stage showed a stronger association with Fat and Starch and a weaker association with Protein and Sugar. Regarding grouting duration, the correlation of T2 and T3 displayed a stronger connection with Fat and Starch, while the correlation of T1 exhibited a stronger association with Protein and Sugar.

### 3.8 Regression analyses of grain filling rate parameters and grain nutritional quality

**Figure 9:**
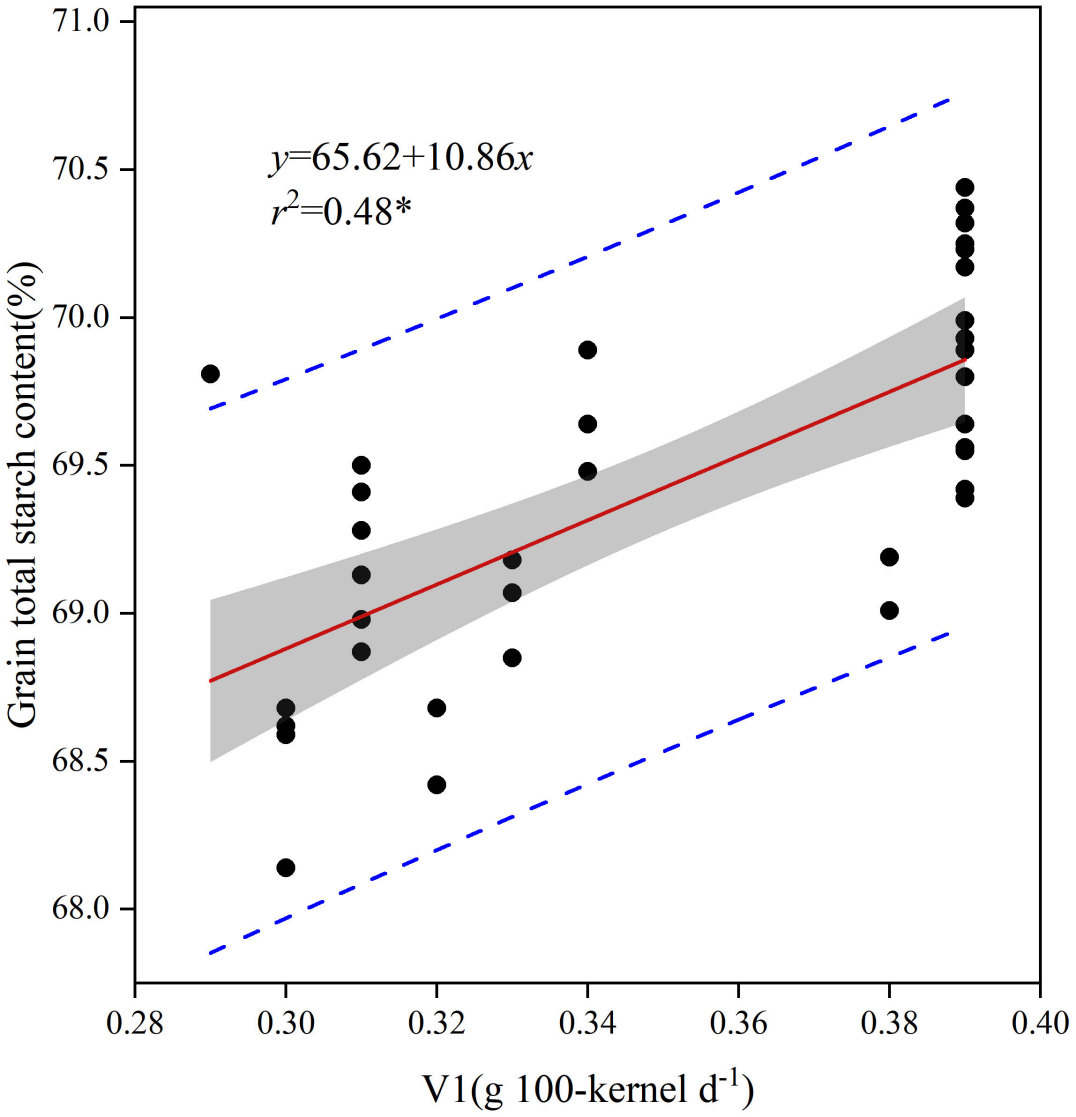
Regression analysis of the grain filling rate parameters and total starch content in grains. *, significant at *p*< 0.05.

Based on the results of the principal component analysis, stepwise regression and multiple linear equations were utilized to fit the grain filling rate (V1, V2, and V3) parameters at each stage with the total starch content and the crude fat content of grain. The analysis revealed that only V1 significantly influenced the total starch content of grain, while V1, V2, and V3 had substantial effects on the crude fat content of grain. The linear function equation for the total grain starch content with V1 from **Figure 9** is represented as *y* = 65.62 + 10.86*x*, showing a positive correlation with the total grain starch content. Additionally, the multivariate linear function equations representing the grain crude fat content and parameters of grain grouting rate at each stage (V1, V2, and V3) were expressed as *y* = 2.99 + 2.34*x1* - 4.21*x2 + *18.43*x3*. Notably, V1 and V3 exhibited a positive correlation with grain crude fat content, while V2 showed a negative correlation (**Table 6**).

**Table 6:** Regression analysis of grain filling rate parameters and grain crude fat content.

Variate	Index	Standard error	*F*-value	Pr > *F*
Intercept	2.99	64.14	89.77	<0.0001
V1 (*x1*)	2.34	1.06	11.53	0.0018
V2 (*x2*)	-4.21	0.74	3.31	0.0782
V3 (*x3*)	18.43	1.20	4.82	0.0355

## 4 Discussion

### 4.1 Effects of ploughing and straw return on grain filling characteristics of different maize varieties

Grain filling is a vital biological process during maize growth and development, significantly influencing the final grain weight and yield. Grouting rate and grouting duration are dynamic traits involved in the formation of grain weight. They respond to the process of grain weight formation, and their interaction collectively determines the magnitude of grain weight. Fang et al. (2020) demonstrated that the filling rate of maize grain governs the accumulation of dry matter in the grain, consequently impacting the yield at harvest. Moreover, they highlighted that employing proper planting practices can enhance the filling rate of the grain. Daynard et al. (1971) observed that extending the duration of grain filling could lead to an increase in 100-grains dry weight. Gasura et al. (2013) proposed that increasing the average grouting rate while extending the active grouting period is more advantageous for maize grain yield. The characteristics of maize grain filling are influenced by the genotype of the variety and the environmental conditions during growth (Wang et al., 2021; Wang et al., 2023b). Subsequently, Deng et al. (2023) discovered that the grouting duration of DH605 exceeded that of ZD958 across various grouting stages, although the grouting rate exhibited inconsistency. Furthermore, Olmedo and Vyn (2021) indicated that a higher nitrogen supply led to an extension in maize grain filling duration regardless of the timing of nitrogen application and planting density but presented inconsistencies in the effect on effective filling rate. In this study, under CK condition, XM6 exhibited a higher peak grouting rate compared to XY696, and the time of its peak grouting rate appeared to be prolonged. However, the changing pattern of each stage’s grouting rate parameter was significantly influenced by the growing season.

In China, soil management and sowing are primarily conducted using small tractors. Without deep plowing, the soil’s surface capacity and resistance to water infiltration increase, posing unfavorable conditions for crop growth. Therefore, the plowing method plays a crucial role in influencing the soil system (Godde et al., 2016; He et al., 2021). Straw return significantly influences soil water, fertilizer, air and heat conditions as well as nutrient accumulation and transformation, impacting crop growth and yield formation. Nevertheless, improper straw return can lead to diminished seeding quality and other adverse effects. Therefore, employing suitable tillage practices along with integrated straw return is not only fundamental for efficient farmland production but also a substantial approach for enhancing soil quality (Li et al., 2022a; Li et al., 2022b). Previous research has indicated that employing suitable tillage practices can enhance maize yield by improving the grain filling characteristics (Zhai et al., 2017; Zhai et al., 2021; Yue et al., 2022). The study revealed that compared to shallow rotary tillage, both the peak grouting rate and the time at which the peak grouting rate occurred exhibited varying degrees of change in all deep tillage and no-tillage treatments. Additionally, the grouting rate and duration differed significantly among different maize varieties in response to the stage of tillage and straw return. Specifically, V1 showed an increase of 1.54%–27.56% in XY696, while V2 and V3 increased by 0.41%–10.42% each in XM6 under all other treatments compared to the shallow rotary tillage. Moreover, for grouting duration, T2 and T3 increased by 1.79%–26.14% for XY696 and 0.11%–21.40% for T1 for XM6 under all other treatments compared to CK.

Numerous prior studies have explored the potential mechanisms through which tillage practices affect dry matter accumulation in maize grains—for instance, subsoiling could promote the grain filling of inferior kernel of summer maize by regulating the soil water content, soil water consumption, and photosynthetic capacity (Zhai et al., 2021). Conservation tillage could promote summer maize photosynthetic capacity and grain filling of inferior kernels by regulating the soil water content and root system morphology (Wang et al., 2021). The SRS (strip rotary tillage without subsoiling) treatment resulted in the highest post-anthesis dry matter accumulation and contribution to grain, and such effect was attributed to the high photosynthetic activity at the later grain filling stage (Chu et al., 2016). In our study, we also found that differences in grain filling rate parameters were significant between the various tillage treatments within the different growing seasons—for example, in 2020, the differences in grain filling rate under different tillage treatments for XY696 were relatively small, whereas in 2021, they significantly increased. We believe that this may be related to meteorological resources during the growth season, as longer periods of sunshine are conducive to photosynthesis in leaves. In other words, superior meteorological conditions during the grain filling process will inevitably lead to an increase in the grain filling rate at the current grain filling stage and will also enhance the regulatory role of some appropriate farming practices on the grain filling rate. Therefore, we believe that, in future research, it may be beneficial to pay moderate attention to the allocation of meteorological resources during the grain filling process and achieve efficient utilization of light and heat resources by adjusting the sowing dates or the application of varieties.

### 4.2 Effect of ploughing and straw return on the nutritional quality of grain of different maize varieties

High crop yields have long been the focus of China’s agricultural development due to the large population, limited land, and persistent scarcity of agricultural products. However, this focus has overshadowed the attention on agricultural product quality. As China’s agricultural production and product availability grow, there will be an increasing demand for higher-quality agricultural products. Crop quality results from both genetic and non-genetic factors. In their study, Wang et al. (2023b) compared high-yielding maize hybrids planted in China over different periods and found that newer varieties exhibited a higher starch content but a lower grain protein content than the older varieties. Our study observed that, under CK treatment, XM6 showed a lower grain crude fat and total soluble sugar content compared to XY696, while no significant difference was found in grain crude protein and total starch content.

Numerous studies and production practices demonstrate that various cultivation measures implemented during the growth and development of crops can significantly influence the yield and quality, with particular emphasis on crop rotation (Smith et al., 2017), planting density (Zheng et al., 2021), fertilization (Dragicěvic et al., 2022), and irrigation (Hussain et al., 2020). In our study, tillage practices had a significant impact on the crude fat and total soluble sugar content of maize grains, with a lesser influence on crude protein and total starch content. We suggest that this outcome may be attributed to the enhancement of soil moisture through tillage practices and the associated ecological benefits. The tillage effects on the examined traits were generally less pronounced than the effects of environment, variety, and input level and could be demonstrated conclusively in interaction with input levels (Šíp et al., 2013). In this context, our study yielded diverse experimental findings—for instance, the total soluble sugar content of grain exhibited the highest variability of 17.27% among varieties and 25.43% among tillage methods. The tillage method and varietal interaction significantly affected the nutrient quality of maize grains (Harish et al., 2022). Upon comparing to CK, the other tillage methods resulted in varying degrees of changes in the nutrient quality components of the grains, with different patterns observed for different varieties. Upon comparing with CK, the other treatments resulted in varying degrees of changes in the nutrient quality components of the grains, with different patterns observed for different varieties. Tillage and straw return treatments led to a reduction in the crude protein and total soluble sugar content of XY696 and XM6 grains, although the decrease in crude protein content was not significant for both varieties; the total soluble sugar content decreased by 4.98%–25.43% and 5.75%–22.42%, respectively. Additionally, the tillage and straw return treatments increased the grain crude fat content of XY696 and XM6 by 4.34%–8.87% and 3.49%–9.67%, respectively. Notably, XM6 exhibited a higher range of increase of grain crude fat content in response to tillage and straw return treatments compared to XY696.

### 4.3 Relationship between grain filling characteristics and nutritional quality

The chemical composition and quality of crop grains are shaped during the accumulation of dry matter and the growth, development, and maturation of organs or tissues. The accumulation of nutrients in cereal crop grains does not occur at a uniform rate; there is a slight increase in dry matter at the beginning of grain filling, the fastest accumulation of dry matter at the milk ripening stage, and a slower increase at the wax ripening stage (Sala et al., 2007). During this stage, there is a significant transport of soluble sugars and non-protein nitrogenous compounds (mainly amino acids) from the plant’s nutrient organs to the reproductive organs, where they are synthesized into starch and proteins in the grains (Yang et al., 2018; Liu et al., 2022b). During grain filling, the majority of sugars originate from the upper leaves, particularly the flag leaves, while protein accumulation is primarily dependent on nitrogenous compounds transported by the nutrient organs, with minimal reliance on materials absorbed by the root system after flowering (Mu et al., 2018; Sun et al., 2022). The quantity and quality of nitrogen-containing compounds undergo further changes as the seed matures (Zhao et al., 2022). In terms of the relationship between grain filling characteristics and nutritional quality, this study identified a strong correlation between grain crude fat and total starch content and the filling rate parameters at all stages (V1, V2, and V3) as well as a high correlation with the duration of the middle and late filling stages (T2 and T3). The differences in the correlation between grain nutrient quality fractions and filling rate parameters suggest variations in the physiological mechanisms involved in synthesizing nutrient quality fractions, leading to different outcomes. Specifically, under normal ripening conditions, protein synthesis primarily occurs at the beginning of the grain filling period, while starch synthesis is limited. As the milk-to-wax ripening period commences, sugar transport to the grain intensifies, and starch synthesis becomes more dominant than protein synthesis. Toward the end of the grain development period, sugar transport to the grain weakens or ceases, while nitrogen inputs continue (Kim et al., 2020). This study also revealed that certain parameters of the grain filling rate, which exhibited a high correlation with grain crude fat and total starch content, played varying roles in influencing the increase of grain crude fat and total starch content, particularly regarding the degree of influence and the regulatory direction.

In our study, we found that differences in grain nutrient quality components among different tillage treatments did not show significant variations between different growth seasons. Taking into consideration the relationship between grain filling characteristics and the content of grain nutrient quality components, we believe that ripening of the grains may have mitigated the impact of grain filling rate and duration at different stages on the component content of grain nutrients. Furthermore, notable studies have successfully predicted maize yield and grain nutritional quality in the absence of crop damage (Guo et al., 2021, Guo et al., 2022, Guo et al., 2023). As such, we aim to incorporate this research aspect into our forthcoming field trials to achieve efficient and high-throughput acquisition of experimental data.

## 5 Conclusion

The appropriate application of tillage measures can significantly affect the 100-grains weight of maize and regulate the content of various nutrient components in grains. The change in grain filling rate significantly influences the variation in the content of nutrient components in grains. Among them, the grain filling rate during the gradual increasing phase is the most important parameter affecting the total starch content of grains, while the grain crude fat content is closely related to the grain filling rates during the gradual increasing and slow increasing phases. The DPR tillage method showed greater improvement in the characteristics of grain filling and grain nutrient quality components, so we suggest that the DPR tillage method should be adopted during maize cultivation in the Western Region of Inner Mongolia. Moreover, we recommend that future research should further investigate the regulation of dry matter accumulation in grains after the long-term implementation of tillage methods. It is advisable to study the effects of proper tillage method implementation on agro-economic aspects.

## Data availability statement

The raw data supporting the conclusions of this article will be made available by the authors, without undue reservation.

## Author contributions

L-QW: Conceptualization, Data curation, Writing – original draft. X-FY: Data curation, Writing – original draft, Writing – review & editing. J-LG: Conceptualization, Writing – review & editing. D-LM: Data curation, Writing – review & editing. H-YL: Writing – original draft. S-PH: Investigation, Writing – review & editing.
